# Muscle fatigue detection and treatment system driven by internet of things

**DOI:** 10.1186/s12911-019-0982-x

**Published:** 2019-12-23

**Authors:** Bin Ma, Chunxiao Li, Zhaolong Wu, Yulong Huang, Ada Chaeli van der Zijp-Tan, Shaobo Tan, Dongqi Li, Ada Fong, Chandan Basetty, Glen M. Borchert, Ryan Benton, Bin Wu, Jingshan Huang

**Affiliations:** 1 Qilu University of Technology (Shandong Academy of Science), Jinan, Shandong China; 2 College of Allied Health Professions, University of South Alabama, Mobile, AL 36608 USA; 3 School of Computing, University of South Alabama, Mobile, AL 36688 USA; 4 College of Medicine, University of South Alabama, Mobile, AL 36688 USA; 5 First Affiliated Hospital, Kunming Medical University, Kunming, Yunnan China

**Keywords:** Wi-fi, Adaptive, PWM, Muscle fatigue, Android

## Abstract

**Background:**

Internet of things is fast becoming the norm in everyday life, and integrating the Internet into medical treatment, which is increasing day by day, is of high utility to both clinical doctors and patients. While there are a number of different health-related problems encountered in daily life, muscle fatigue is a common problem encountered by many.

**Methods:**

To facilitate muscle fatigue detection, a pulse width modulation (PWM) and ESP8266-based fatigue detection and recovery system is introduced in this paper to help alleviate muscle fatigue. The ESP8266 is employed as the main controller and communicator, and PWM technology is employed to achieve adaptive muscle recovery. Muscle fatigue can be detected by surface electromyography signals and monitored in real-time via a wireless network.

**Results:**

With the help of the proposed system, human muscle fatigue status can be monitored in real-time, and the recovery vibration motor status can be optimized according to muscle activity state.

**Discussion:**

Environmental factors had little effect on the response time and accuracy of the system, and the response time was stable between 1 and 2 s. As indicated by the consistent change of digital value, muscle fatigue was clearly diminished using this system.

**Conclusions:**

Experiments show that environmental factors have little effect on the response time and accuracy of the system. The response time is stably between 1 and 2 s, and, as indicated by the consistent change of digital value, our systems clearly diminishes muscle fatigue. Additionally, the experimental results show that the proposed system requires minimal power and is both sensitive and stable.

## Background

The Internet of Things (IoT) refers to interconnecting computing devices embedded in everyday objects and enabling them to transfer and receive data over the internet. The IoT is quickly spreading to virtually all aspects of people’s lives including the integration of the IoT into medical treatment which is highly beneficial to both doctors and patients and increasing everyday [1]. Muscular fatigue is defined as a reduction in a muscle’s force-generating capacity due to exercise [2]. Muscle fatigue due to repetition incurs a loss of functional ability [3] and, as evidenced in sports, directly relates to muscular strength [4]. Massages can help alleviate muscle fatigue [5] through improving local blood circulation and nutrition and helping to accelerate lactic acid discharge.

Muscle fatigue routinely occurs in daily life and as the detection and evaluation of musclar fatigue clearly has practical significance has become a highly active area of research. In 2010, D. Tkach [6] examined time domain stability with surface electromyography (sEMG) pattern recognition and successfully extracted frequency domains and features from these time domain analyses which facilitated more efficient evaluation of muscle fatigue. That same year, Wang Kui [7] similarly utilized a combination of frequency domain, time domain, and time-frequency allowing a more comprehensive evaluation of muscle fatigue. In 2011, Wang Fenjuan [8] described a muscle fatigue detection system integrating an ARM microcontroller core combined with a AgCl surface electrode allowing for the effective identification of sEMG signal s through both digital and analog filters. Notably, this constituted the first time a micro-controller unit (MCU) was incorporated into a muscle fatigue detection system; that said, although the MCU provided a suitable baseline signal for research, its hardware was complex and operation cumbersome, and ultimately proving unsuitable for clinical application. Also in 2011, Wan Sha [9] created a multi-channel sEMG detection system utilizing LabVIEW. While an improvement over exisiting technologies since the sEMG signal could be acquired in real time and visualized on a personal computer (PC), LabView carried significant delays when switching between channels. More recently, in 2017, Zhu Anyang [10] generated a STM32-based sEMG acquisition system in which the sEMG signal is transmitted through a USB interface to the host computer then analyzed and processed directly. Importantly, however, while each of these [8–10] sEMG signal detection systems were innovative in the time, none of these have intelligent terminals, significantly hindering their usability.

Of note, in 2014, Richer [11] developed a real-time electromyogram (EMG) and electrocardiogram (ECG) that could calculate the real time heart rate and rhythm of cyclists on mobile Android devices. In 2016, Widasari [12] proposed a similar smartphone-based sEMG monitoring system capable of alerting users before muscle fatigue occurs. Most recently, in 2018, Yamaguchi [13] created a wearable sEMG monitoring device that recognizes bruxism via analog values obtained from the masseter sEMG signal then alerts users. Although these [11–13] sEMG monitoring systems have been designed with intelligent terminals that successfully integrate sEMG signal detection with user wear, their functions are singularly focused on specific, isolated tasks.

That said, this report describes a highly innovative muscle fatigue detection and treatment platform that seamlessly combines the advantages of previous systems in order to integrate sEMG signal detection and human muscle fatigue release into a comprehensive, user-friendly IoT system. In this platform, sEMG signals are acquired using a myoelectric sensor then sent to an ESP8266 where they are converted from analog to digital sEMG signals. The digital value is then used to control an adaptive vibrating motor to help relieve muscle fatigue. The application (APP) charged with monitoring and controlling unit functions can also be used to manually turn vibrations on or off, making the unit more user-friendly.

In large part, this manuscript constitutes an extension of our previously published [14] initial description of our system. That said, this report describes upgrades to our platform including the addition of an infra-red transducer that can determine when someone is using the system, and also describes our system software in significantly greater detail.

## Methods

### Overall system design

The complete system is composed of a power supply, an EMG transducer, an infra-red transducer, an ESP8266 Wi-Fi module, vibration motors, and a motor drive module. The motor drive module is used to control the vibration motors. The ESP8266 wireless Wi-Fi module is both MCU and Wi-Fi transmitter/receiver for the system and charged with using minimal power to process sensor data and relay signals.

**Fig. 1:**
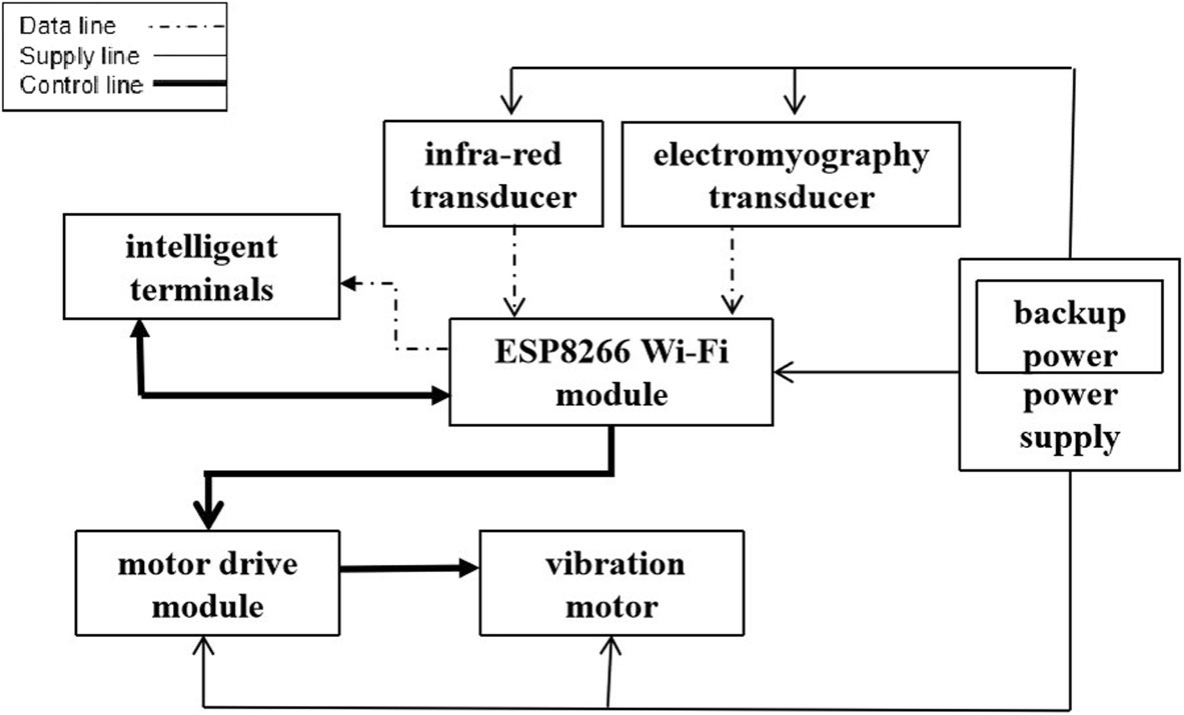
Diagram of the overall system structure

The power supply offers 3.3 V, 5 V, and 12 V direct current (DC) positive and negative poles. The infra-red transducer detects the infra-red signal to determine whether someone is using the system. The EMG transducer determines muscle activation through potential then transmits an EMG pulse signal. An ESP8266 serves as the sole communicator and processor processing monitored data retrieved from the sensor. One of 3 gears can be employed by the vibration motor depending on the extent of muscle activation represented as a digital value. Soft access point (AP) mode is adopted when the ESP8266 Wi-Fi module communicates during which the ESP8266 serves as a wireless access point. The mobile device APP functions as a station to allow connection to the Wi-Fi module issued hotspot. Our system utilizes the TCP/IP protocol suite (TCP/IP) for transmitting data through socket matching. Overall system structure, including the representation and connection of hardware entities, is depicted in Fig. 1. The overall structure provides a basis for classifying equipment inside the 3-tier architecture of IoT.

**Fig. 2:**
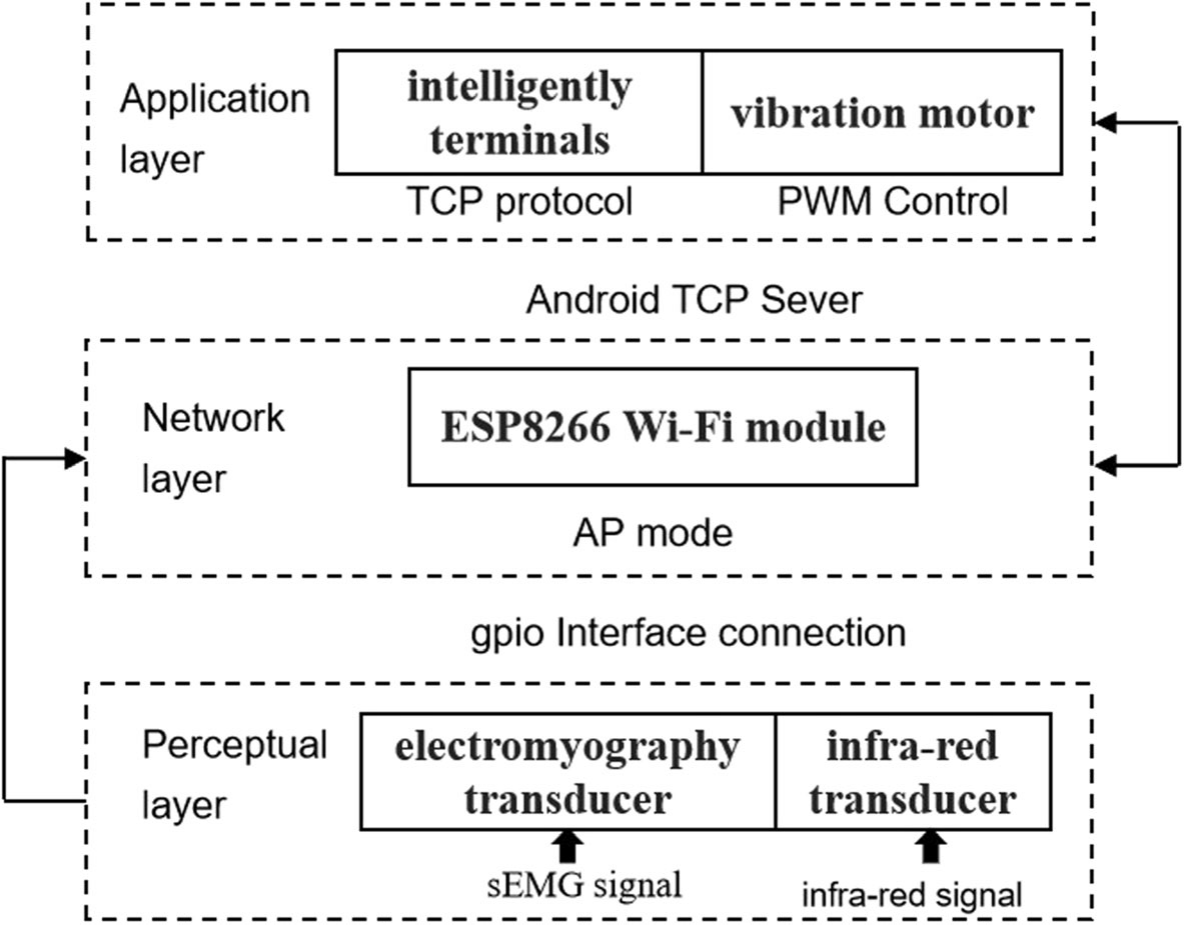
The architecture of three-tier IoT

Our adaptive system was constructed to deliver real time muscle information. IoT architecture consists of 3 parts: the perceptual bottom-level layer, the network middle-level layer, and the upper level application layer [15]. Each of these layers is supported by corresponding systems and middleware [16], as illustrated in Fig. 2. In this system, the perceptual layer consists of an EMG transducer that receives the EMG signal and an infra-red transducer that detects the infra-red signal. The network layer processes and sends the data obtained by the ESP8266 to intelligent mobile terminals. At the application layer, the information obtained by the perceptual layer is processed and displayed. The intelligent terminal has control and management functions.

### Design of the perceptual layer

In this system, the perceptual layer consists of an EMG transducer that receives the EMG signal.

#### Physiological basis of sEMG signal generation

**Fig. 3:**
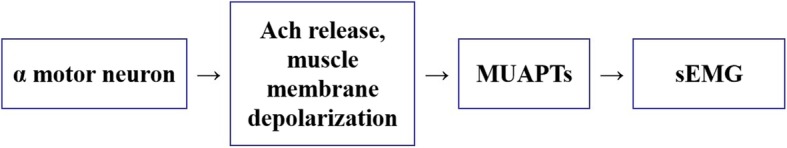
The EMG signal formation process

sEMG signal is a one-dimensional time-series voltage signal superimposed on the surface of the skin by the action potential sequence generated by the motor unit when the muscle reaches the excited state under the control of the nervous system [17]. The EMG signal formation process is shown in Fig. 3.

#### EMG transducer

**Fig. 4:**
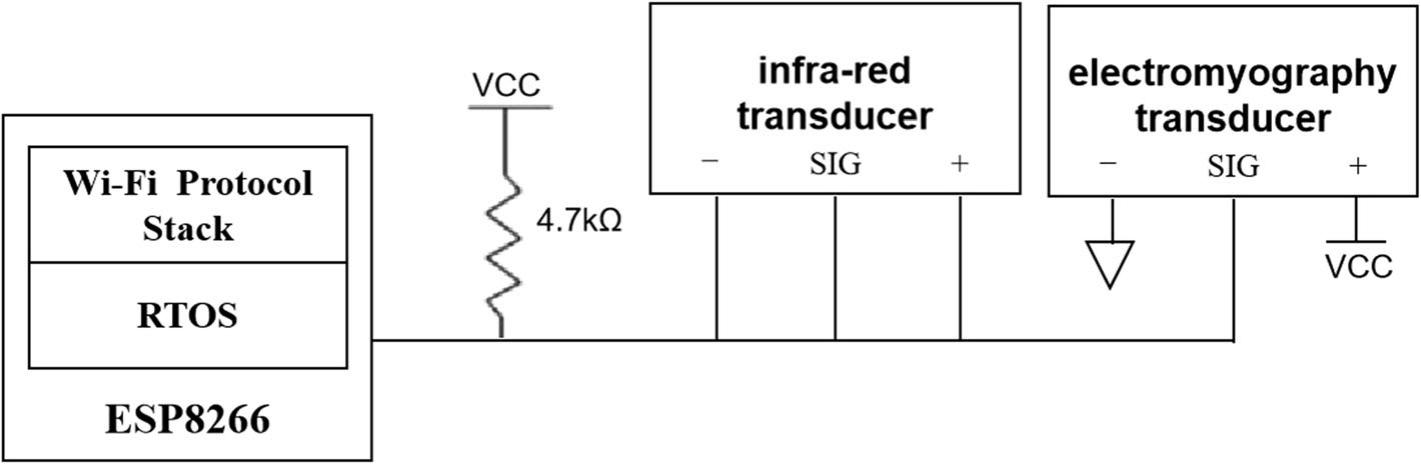
Schematic diagram connecting the ESP8266 and EMG transducer

The transducer measures the real world degree of muscle activation through measuring electrical potential. The sEMG transducer can output both raw and pulse EMG signals. The system does not relay the original sEMG signal rather, it outputs, through the transducer, an amplified, rectified, and integrated pulse signal. Signal filtering occurs in the output pulse signal, with both low and high pass included. After signal amplification, the ESP8266 performs analog-to-digital conversion, and the digital EMG signal can then be intuitively and easily detected. A schematic diagram of the connection among the ESP8266, the EMG transducer, and the infra-red transducer is shown in Fig. 4.

### Design of the network layer

In this system, the network layer processes and sends the data obtained by the ESP8266 to intelligent mobile terminals.

#### Selection of the ESP8266 mode

**Fig. 5:**
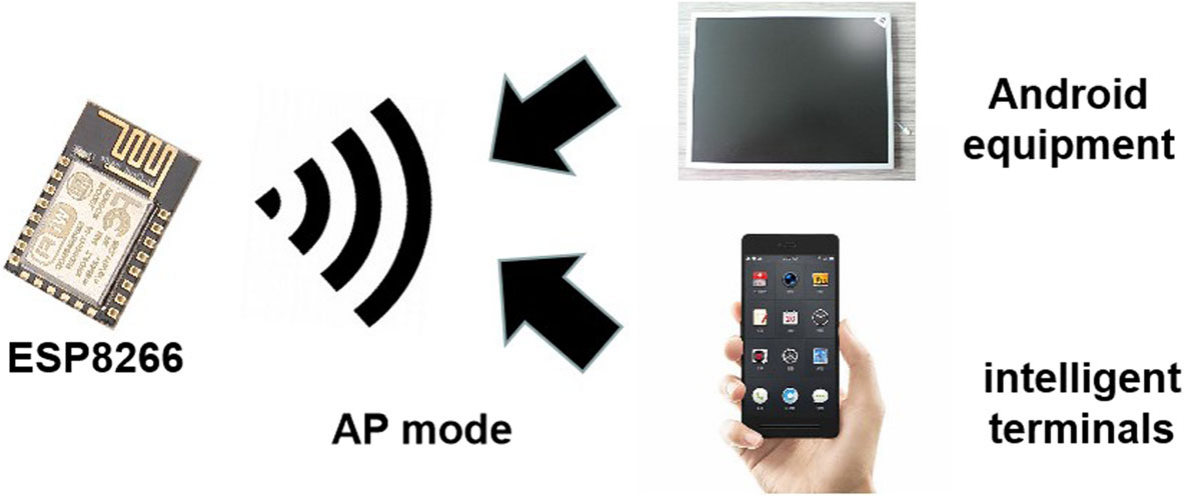
ESP8266 in AP mode

The most integrated Wi-Fi chip, ESP8266, is based on the library for WWW access in Perl (LWP) protocol, and offers three modes from which to select: AP, Station (STA), and AP + STA. In the AP mode, the Wi-Fi module is a wireless access point that functions as a router, and the chip is the creator of a wireless network. In the STA mode, it connects to the terminal as an AP and does not accept wireless access itself. AP + STA is owned by both modes and can be used as an AP or a station to connect to other hotspots. In our system, the ESP8266 is utilized in AP mode, and its operation diagram is shown in Fig. 5.

#### ESP8266 software development kit (SDK)

ESP8266 is typically employed in two ways. One way is to select the working parameters and modes of the chip using the attention (AT) command, typically requiring another MCU. When employed in this manner, the ESP8266 must communicate with the MCU via the serial port. In contrast, the other way for this chip to be utilized is in secondary development of an official SDK, not only implementing data transmission but also processing sensor data collection. However, the ESP8266 chip has a built-in 32-bit central processing unit (CPU) with significant storage and on-chip processing capabilities and can be alternatively utilized to control sensors and applications through general-purpose input output (GPIO). When the ESP8266 chip is the only processor in the system, it can be started directly from a flash drive using minimal memory resources.

The ESP8266 in this system is set to operate in the second way, which allows it operates independently. Since the entire system is designed to minimize the occupied printed circuit board (PCB) space, the circuit design is very simple, along with achieved low-power control.

#### Adaptive pulse width modulation (PWM) control

Our system has 2 operating modes: a fully automatic mode and a semi-automatic mode. Users can select a mode via the intelligent mobile terminal. When in the fully automatic mode, the system works according to a preset program. Vibration motor vibration strength changes in sync with indiviual changes of the electromyographic signal. Vibration strength of the motor is calculated by the duty of PWM [18], and the following Table 1 gives the corresponding relationship. In semi-automatic mode, users can control, through the intelligent mobile terminal, if the vibration motor vibrates or not. When the “Close” button is selected on the APP side, the vibration motor stops working immediately and no longer changes with the EMG signal.

**Table 1:** PWM Gears

Gears	EMG signal digital value	The duty of PWM	Vibration strength
1	< 200	222	weak
2	200–500	2222	normal
3	> 500	18,000	strong

### Design of the application layer

The application layer has two major sets of functionality. First, the application layer is responsible for processing and displaying the information obtained from the perceptual layer. Second, the application layer, realized through the intelligent terminal, is responsible for the system’s control and management functions.

The Android terminal consists of a server and a client [17], and was implemented using the Java language and the development environment (IDE) of Android studio [19]. The software design primarily focused upon network communication, data transmission, and the man-machine interface [20]. During the process of software development, the full degree of integration between software design and hardware was comprehensively considered [21], and the features of reliability and modularity fully employed to enhance the intelligence of the overall system [22, 23].

A design element of the system is both the Wi-Fi module and intelligent mobile terminal communicate via TCP/IP protocol. As a result, users can utilize the intelligent mobile terminal for connection to the wireless hotspots provided by the Wi-Fi module for accessing the muscles’ rehabilitation management control via the APP.

**Fig. 6:**
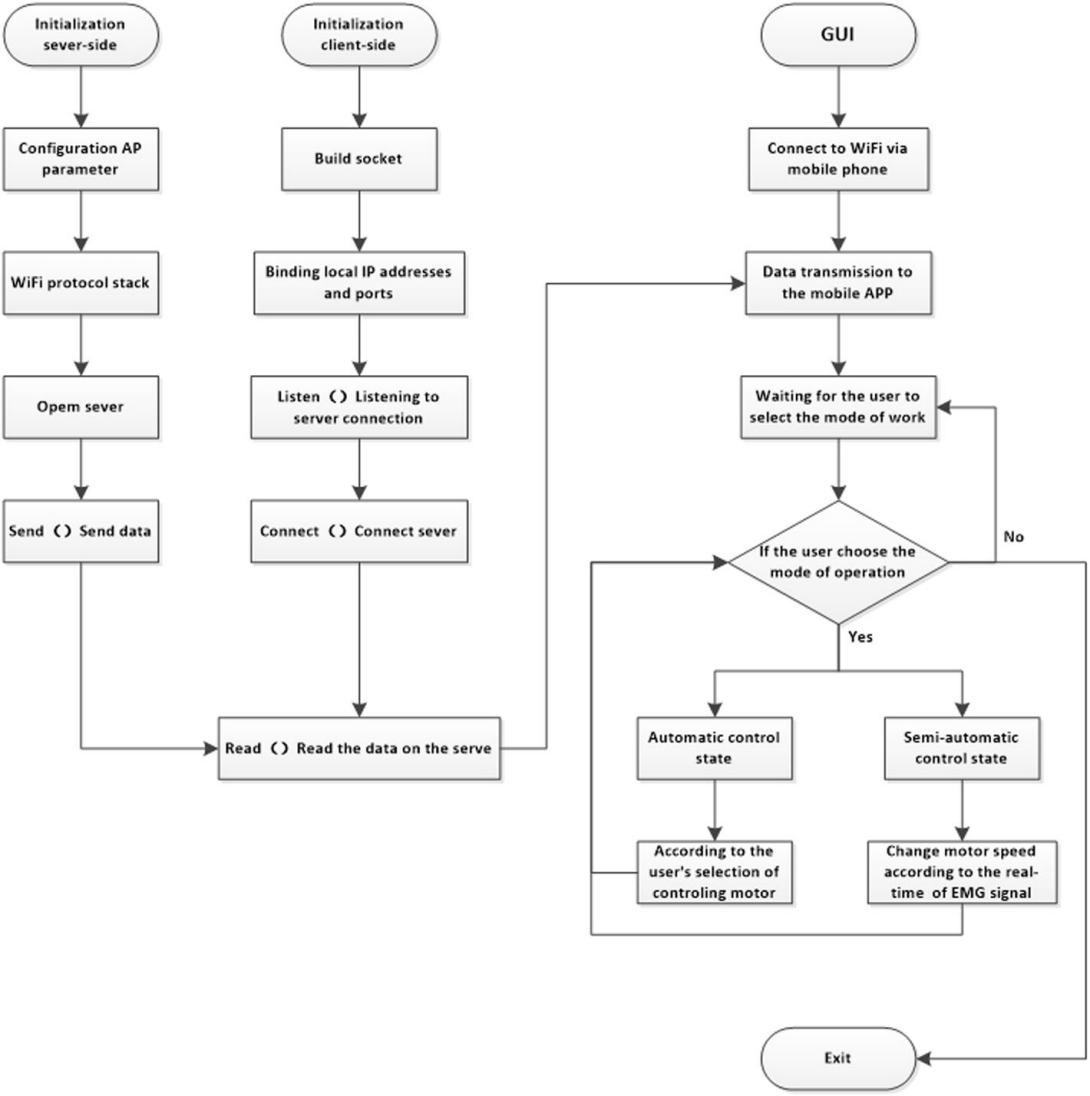
The structure of software design

As mentioned earlier, the software design of the system (see Fig. 6) is specifically implemented in two major components: the server and the client. First, the ESP8266 is developed into server side platform, and a multi task scheduling mechanism is assumed. The ESP8266 collects data from the perceptual layer and sends it to the client. In the process of ESP8266 SDK’s development, the server’s Internet protocol (IP) address and port are set, and the service set identifier (SSID) and password are set. Data transmission between the client and the server occur through the socket for communication between the two sides. Second, an Android-based APP is used as a client for the system to set an IP address to port the APP for receiving data implementing control and detection functions of the mobile device.

To achieve network communication, the Mina framework was incorporated. Apache Mina Server constitutes a communication framework based on the TCP/IP, UDP/IP protocol suite (UDP/IP) stacks. It not only assists developers with quickly creating high-performance, expandable network communication applications, but it also provides an event driven, asynchronous operation model. The Mina framework also solves common problems including, but not limited to, network management software input/output (I/O) transmission random bursts and numerous client connections. Additionally, developers can also use it to easily create complex tasks such as low-level I/O and thread concurrency. Hence, by using the Mina framework, we were able to implement efficient and robust data transmission between our proposed server and client.

When first contacted by a client, the server first configures AP parameters, like IP addresses and server ports, through the TCP/IP protocol. These are then written into the Wi-Fi protocol to establish a connection with the client, and client and server socket channels simultaneously generated to ensure data transmission. Following this, the server will listen to the client’s request and call the accept method to finally establish the connection. After the connection with the client is established successfully, data transmission will be performed using the transceiver function according to the requirements of the control flow.

The Android application is a mobile device system that provides operations through the user interface (UI). Following common practice, the Android application development has been divided into UI design and activity function implementation. At first, the developer performs UI design by coding the extensible markup language (XML) file and then uses the layout combined with a multi-layout manager interface to make the design more aesthetically pleasing. Finally, according to the layout of the user interface, the main function of the Android application is realized by writing the MainActivity file.

## Results and discussion

### Experimental design

Our experimental design was as follows. Ten groups of randomly aged men (10 men in each group, 100 men in total) were examined. The average physical condition of the ten groups is shown in Table 2. No participants reported any musculoskeletal or neurological diseases. The right biceps brachii was examined by placing one EMG transducer electrode on skin above the muscle center and another along the extended direction of the same biceps brachii. A reference electrode was also placed in a position without underlying muscle.

**Table 2:** Experimental Subject Information

Test subject	Gender	Age	Body height	Body weight
S1	male	18	178	66
S2	male	20	175	68
S3	male	25	174	81
S4	male	30	173	76
S5	male	35	171	71
S6	male	38	175	70
S7	male	40	170	79
S8	male	45	173	76
S9	male	50	170	72
S10	male	60	168	64

### Indoor experiment

**Fig. 7:**
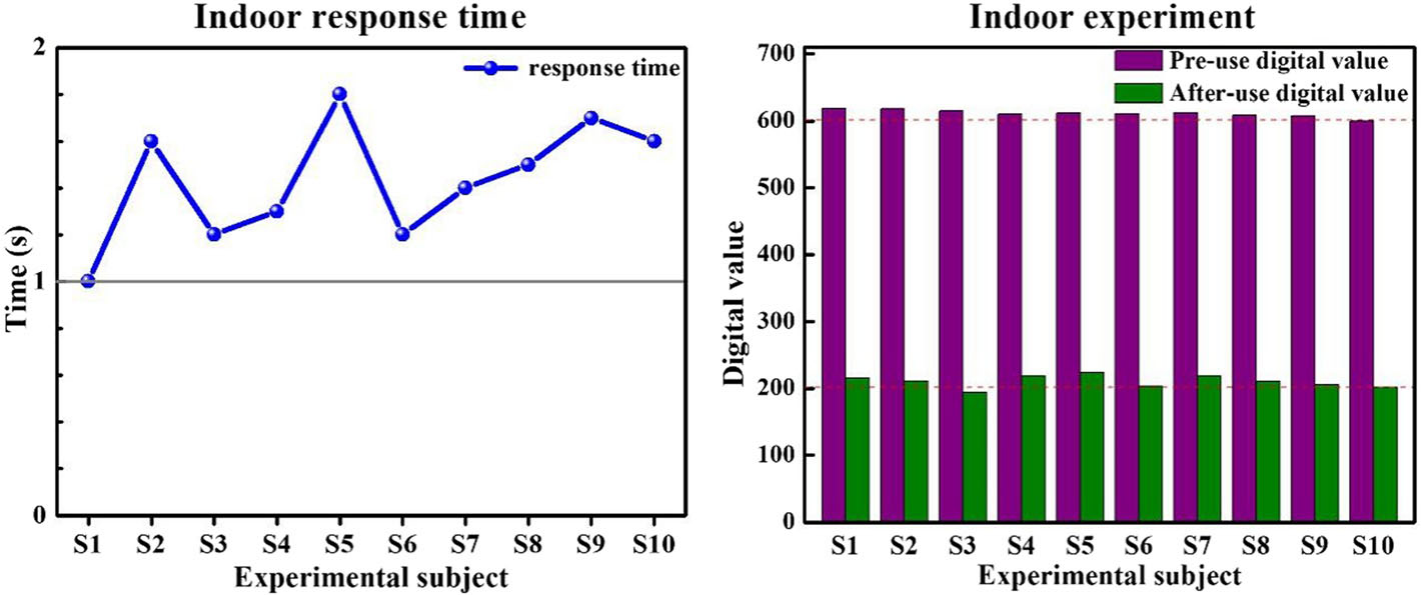
Experimental results in an indoor environment

Prior to testing, identical massagers were used to relax the biceps brachii for 2 min. All testers stood in a standing position with arms naturally hanging by their sides and their right hand holding a 1.5 kg dumbbell. Testers then repeated identical wrist flexion and extension exercises 10 times. Experimental results represent the average of 10 repeated series of 10 flexions and extensions. Experimental testing was performed indoors (see Fig. 7) at 26 °C.

### Outdoor experiment

**Fig. 8:**
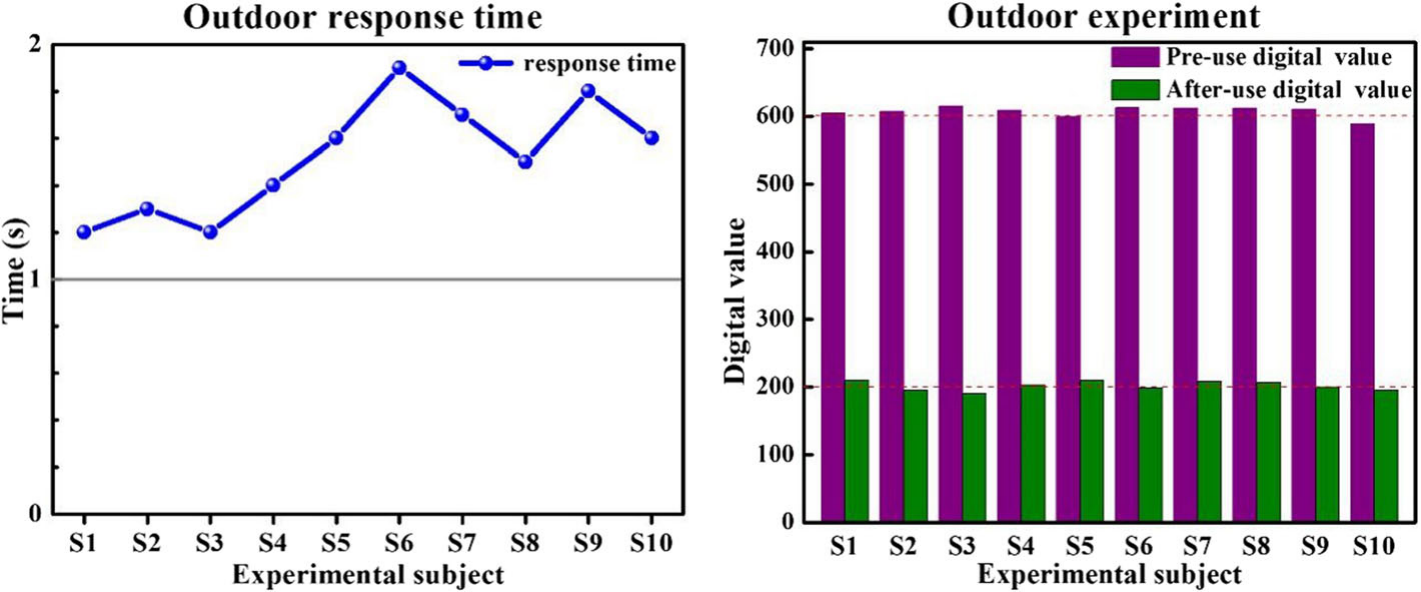
Experimental results in an outdoor environment

In order to better evaluate the anti-jamming index and anti-jamming performance of our adaptive system in a more realistic environment, we also conducted a group of identical interference tests outdoors (see Fig. 8). Outdoor experiments were performed at 6 °C with only minimal wind.

### Effectiveness and stability of the system

Our experiments clearly indicate that environmental factors have little effect on the accuracy and response time of our system. Combined with Tables 3 and 4, it is apparent that response time was stable between 1 and 2 s. As indicated through the consistent change of digital value, muscle fatigue was clearly diminished when employing this system.

**Table 3:** Experimental Data in Indoor Environment

Experimental subject	Response time (s)	Digital value before use	Digital value after use	Corresponding gear shift
S1	1.0	619	216	3 → 2
S2	1.6	618	211	3 → 2
S3	1.2	615	194	3 → 1
S4	1.3	610	218	3 → 2
S5	1.8	612	224	3 → 2
S6	1.2	611	203	3 → 2
S7	1.4	613	218	3 → 2
S8	1.5	609	210	3 → 2
S9	1.7	608	205	3 → 2
S10	1.6	600	201	3 → 2

**Table 4:** Experimental Data in Outdoor Environment

Experimental subject	Response time (s)	Digital value before use	Digital value after use	Corresponding gear shift
S1	1.2	605	209	3 → 2
S2	1.3	607	195	3 → 1
S3	1.2	615	190	3 → 1
S4	1.4	609	202	3 → 2
S5	1.6	599	209	3 → 2
S6	1.9	613	198	3 → 1
S7	1.7	612	208	3 → 2
S8	1.5	612	206	3 → 2
S9	1.8	610	199	3 → 1
S10	1.6	589	195	3 → 1

## Conclusions

Muscle fatigue is a problem encountered quite frequently in our daily life. To assist with alleviating muscle fatigue, we designed an adaptive muscle fatigue detection and recovery system based on PWM and ESP8266. Note that ESP8266 is not only a Wi-Fi adapter, but also a processor that can run independently. Our system’s main function is to prevent the muscle fatigue through the adoption of adaptive technology. Experiments on system performance have indicated that our system has low power consumption and simple hardware composition. Furthermore, our system can be effectively employed to prevent muscle fatigue, has simple and stable operations, and well meets our design requirements. In short, the system introduced in this paper exhibits an innovative application of the IoT technology in modern medicine, with clear potential for an effective management of muscle fatigue.

In our future work, we will explore how to further improve the system performance by integrate additional sensors so as to determine muscle fatigue in a more comprehensive manner.

## Data Availability

Data sharing is not applicable to this article as no datasets were generated or analyzed during the current study.

## Abbreviations

A/D: Analog to Digital
AP: Access Point
APP: Application
AT: Attention
CPU: Central Processing Unit
DC: Direct Current
ECG: Electrocardiogram
EMG: Electromyogram
GPIO: General-Purpose Input Output
I/O: Input/Output
IDE: Integrated Development Environment
IoT: Internet of Things
IP: Internet Protocol
LWP: Library for WWW access in Perl
MCU: Micro-Controller Unit
PC: Personal Computer
PCB: Printed Circuit Board
PWM: Pulse width Modulation
SDK: Software Development Kit
sEMG: surface Electromyography
SSID: Service Set Identifier
